# Increased Radiation-Associated T-Cell Infiltration in Recurrent IDH-Mutant Glioma

**DOI:** 10.3390/ijms21207801

**Published:** 2020-10-21

**Authors:** Anastasia Makarevic, Carmen Rapp, Steffen Dettling, David Reuss, Christine Jungk, Amir Abdollahi, Andreas von Deimling, Andreas Unterberg, Christel Herold-Mende, Rolf Warta

**Affiliations:** 1 Division of Experimental Neurosurgery, Department of Neurosurgery, University Hospital of Heidelberg, 69120 Heidelberg, Germany; anastasia_makarevic@web.de (A.M.); Carmen_Rapp@gmx.de (C.R.); steffen.dettling@gmail.com (S.D.); christine.jungk@med.uni-heidelberg.de (C.J.); andreas.unterberg@med.uni-heidelberg.de (A.U.); Christel.Herold-Mende@med.uni-heidelberg.de (C.H.-M.); 2 German Cancer Consortium (DKTK), 69120 Heidelberg, Germany; David.Reuss@med.uni-heidelberg.de (D.R.); a.amir@dkfz.de (A.A.); Andreas.vonDeimling@med.uni-heidelberg.de (A.v.D.); 3 Clinical Cooperation Unit Neuropathology, German Cancer Research Center (DKFZ), 69120 Heidelberg, Germany; 4 Department of Neuropathology, Institute of Pathology, Ruprecht-Karls-University, 69120 Heidelberg, Germany; 5 Department of Neurosurgery, University Hospital of Heidelberg, 69120 Heidelberg, Germany; 6 Molecular and Translational Radiation Oncology, National Center for Tumor Diseases (NCT), Heidelberg University Hospital and German Cancer Research Center (DKFZ), 69120 Heidelberg, Germany

**Keywords:** lower-grade glioma, T-cell infiltration, TissueFAXS, primary tumors, recurrent tumors, radiotherapy

## Abstract

Most gliomas are associated with a fatal prognosis and remain incurable because of their infiltrative growth. Consequently, the addition of immunotherapy to conventional therapy may improve patient outcomes. Here, we analyzed T-cell infiltration and, therefore, a major prerequisite for successful immunotherapy in a series of primary (*n* = 78) and recurrent (*n* = 66) isocitrate dehydrogenase (IDH)-mutant glioma and their changes following treatment with radio- and/or chemotherapy. After multicolor immunofluorescence staining, T cells were counted in entire tumor sections using a software-based setup. Newly diagnosed diffuse IDH-mutant gliomas displayed a median T-cell infiltration of 0.99 T cells/mm^2^ (range: 0–48.97 CD3^+^ T cells/mm^2^), which was about two-fold increased for CD3^+^, helper, and cytotoxic T cells in recurrent glioma. Furthermore, T-cell infiltration of recurrent tumors was associated with the type of adjuvant treatment of the primary tumor. Interestingly, only glioma patients solely receiving radiotherapy presented consistently with increased T-cell infiltration in their recurrent tumors. This was confirmed in a subset of 27 matched pairs. In conclusion, differences in the T-cell infiltration of primary and recurrent gliomas were demonstrated, and evidence was provided for a beneficial long-term effect on T-cell infiltration upon treatment with radiotherapy.

## 1. Introduction

Glioma represents one of the most frequent malignant primary brain tumors [1]. The World Health Organization (WHO) classifies gliomas into four ascending grades of malignancy based on histopathological criteria [2]. Diffuse lower-grade glioma (LGG), which comprises astrocytoma and oligodendroglioma of WHO grades II and III, accounts for approximately one-third of all gliomas [1]. While the more common astrocytoma shows a median overall survival of 8.1 and 5.6 years for WHO grade II and III tumors, respectively, oligodendrogliomas exhibit a more favorable prognosis (12.2 and 6.3 years for WHO grade II and III, respectively) [3]. During the last two decades, genetic analyses identified point mutation within the gene encoding the enzyme isocitrate dehydrogenase (IDH) to be an early event in gliomagenesis, thus showing an especially high prevalence in LGG [4,5]. Since this mutation is also significantly associated with younger age at diagnosis and better overall survival, it was included in the latest WHO classification [2,3]. These results suggest that IDH-mutant (IDH^mut^) and IDH-wildtype (IDH^wt^) gliomas are indeed different tumor entities with a distinct tumor biology [6].

Although LGG belongs to a group of rather slow-growing tumors of the central nervous system, they are still considered incurable due to their highly infiltrative nature [2]. The current conventional treatment options range from surgical resection to radiochemotherapy [7]. However, recurrence frequently occurs mostly due to tumor cells remaining in the brain parenchyma posttreatment [8]. Hence, new therapeutic strategies are required to eliminate such cells to lower the relapse rate. On this account, immunotherapy has already emerged as a novel treatment modality for various types of cancer [9]. Promising immunotherapeutic approaches use the capability of T cells to initiate a specific and powerful antitumor immune response [10]. Since the ratio between malignant target cells and cytotoxic T cells was shown to be crucial for an effective tumor cell lysis, it is not surprising that the amount of tumor-infiltrating lymphocytes (TILs) positively correlates with better patient survival in several solid tumors [11,12]. Similarly, the level of intratumoral CD8^+^ cytotoxic T lymphocytes is associated with a better prognosis of particularly primary glioblastoma (GBM) patients and increases with the degree of glioma malignancy [11,13]. Moreover, CD8^+^ T cells, CD4^+^ T helper cells, and FoxP3^+^ regulatory T cells (T_regs_) infiltrate gliomas, with the latter conveying immunosuppressive functions and therefore diminishing an effective antitumor immune response [14].

In addition to the relatively low mutation rate of gliomas and the resulting sparse appearance of neoantigens, the blood–brain barrier widely shields brain tissue from surveillance by the peripheral immune system, which might also impair the efficacy of immunotherapy for glioma [15]. Until now, most research has been conducted on the tumor biology of GBM, the most aggressive type of glioma. For this reason, little is known about the appearance of immune cells in LGGs and especially IDH^mut^ LGGs ([6,16,17,18], Table S1). However, the growing knowledge of the heterogeneous biology of this group provides new immunotherapeutic possibilities of intervention. Simultaneously, it highlights the importance of examining IDH^mut^ glioma separately and of shedding more light onto immunological changes during the course of disease.

In the present study, we analyzed the number of TILs in IDH^mut^ glioma by multicolor immunofluorescence staining in a large series of newly diagnosed and recurrent tumors. We observed a two-fold increased T-cell infiltration in recurrent glioma, which was especially pronounced in patients previously treated with radiotherapy. 

## 2. Results

### 2.1. Characterization of the Glioma Study Samples

To study T-cell infiltration in gliomas, specimens were categorized according to the IDH mutation status into IDH^mut^ (*n* = 144; Table 1 and Table 2 and Table S2) and IDH^wt^ (*n* = 14; Table S3) samples. Primary IDH^mut^ gliomas (*n* = 78) comprised 44 WHO grade II and 34 WHO grade III gliomas (Table 1). The mean age at initial diagnosis was 43.2 years (range 20.8−80.1). The recurrent IDH^mut^ glioma group (*n* = 66) consisted of 15 WHO grade II and 37 WHO grade III gliomas as well as 14 WHO grade IV secondary glioblastomas (Table 2). The average age at current diagnosis was 42.2 years (range 20.8–71.5). All primary IDH^mut^ gliomas were treatment-naïve, while recurrent tumors often had a diverse treatment history for the primary tumors. Treatment included radiotherapy applied as a monotherapy (*n* = 21), chemotherapy (*n* = 8), and combined radiochemotherapy (*n* = 14). Twenty-three patients with recurrent glioma received surgery as a single treatment for their primary tumor. Finally, the subset of IDH^wt^ glioma consisted of two WHO grade II astrocytoma, seven WHO grade III astrocytoma, and four WHO grade IV glioblastoma, with a mean age of 50.6 years (range 15.9–75.7; Table S3).

### 2.2. Quantification of T-Cell Infiltration

**Figure 1:**
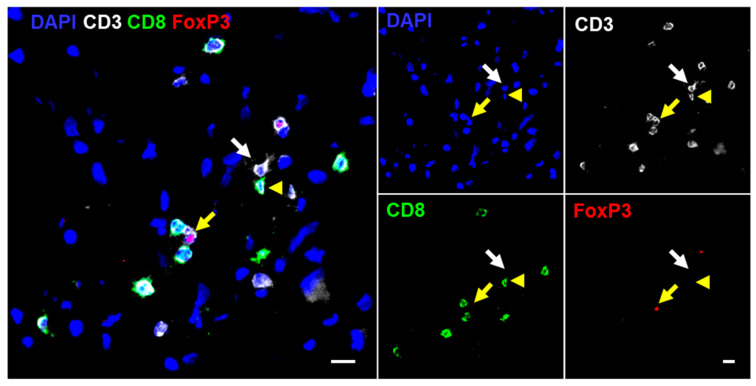
Immunofluorescence staining of CD3, CD8, and FoxP3: detection of T-cell subsets by multicolor immunofluorescence staining is visualized by an overlay (left) and single fluorescence images (right). The white arrow points towards T helper cells (CD3^+^CD8^−^FOXP3^−^), the yellow arrowhead points towards cytotoxic T cells (CD3^+^CD8^+^FOXP3^−^), and the yellow arrow points towards a regulatory T cell (CD3^+^CD8^−^FOXP3^+^). Scale bar = 10 µm.

To quantify TILs in glioma specimens, multicolor immunofluorescence staining was performed, followed by software-based image analysis. DAPI co-staining for the identification of nucleated cells enabled reliable quantification of T-cell marker-expressing cells. T cells were recognized and subclassified based on staining of the markers CD3, CD8, and FoxP3 (Figure 1). TissueQuest software was used to quantify marker-positive cells and to perform flow cytometry-like hierarchical gating, facilitating the distinction of cytotoxic T cells (CD3^+^CD8^+^FOXP3^−^), T helper cells (CD3^+^CD8^−^FOXP3^−^), and regulatory T cells (CD3^+^CD8^−^FOXP3^+^) (Figure S1). While immunofluorescent labeling of CD3 and CD8 was measured at the T-cell surface, staining of the transcription factor FoxP3 was restricted to the nucleus. To account for the expected low T-cell infiltration rate in gliomas and the intratumoral heterogeneity, whole tissue sections with a mean area of 14.34 mm^2^ were scanned and analyzed.

### 2.3. Increased T-Cell Infiltration in Primary IDH^wt^ Versus IDH^mut^ Glioma

When comparing overall T-cell infiltration (CD3^+^) in primary glioma with and without the most common IDH1(R132H) mutation (Table 1 and Table S3), we observed a significantly higher T-cell infiltration in IDH^wt^ compared to IDH^mut^ astrocytoma (Figure S2a), which is in accordance with current literature [6]. In contrast, apart from a nonsignificant increase in oligodendrogliomas, T-cell infiltration in astrocytoma and oligodendroglioma with a proven IDH mutation was rather comparable. However, there was a nonsignificant trend towards higher numbers of cytotoxic and T helper cells in IDH^wt^ compared to IDH^mut^ astrocytoma (Figure S2b,c). Regulatory T cells were rarely detected and comparable across the different glioma groups (Figure S2d). Due to the small sample size of IDH^wt^ gliomas, a more detailed analysis, such as the effect of the WHO grade, was not possible (Table S3).

### 2.4. Differential T-Cell Infiltration in Primary IDH^mut^ Glioma Subtypes

**Figure 2:**
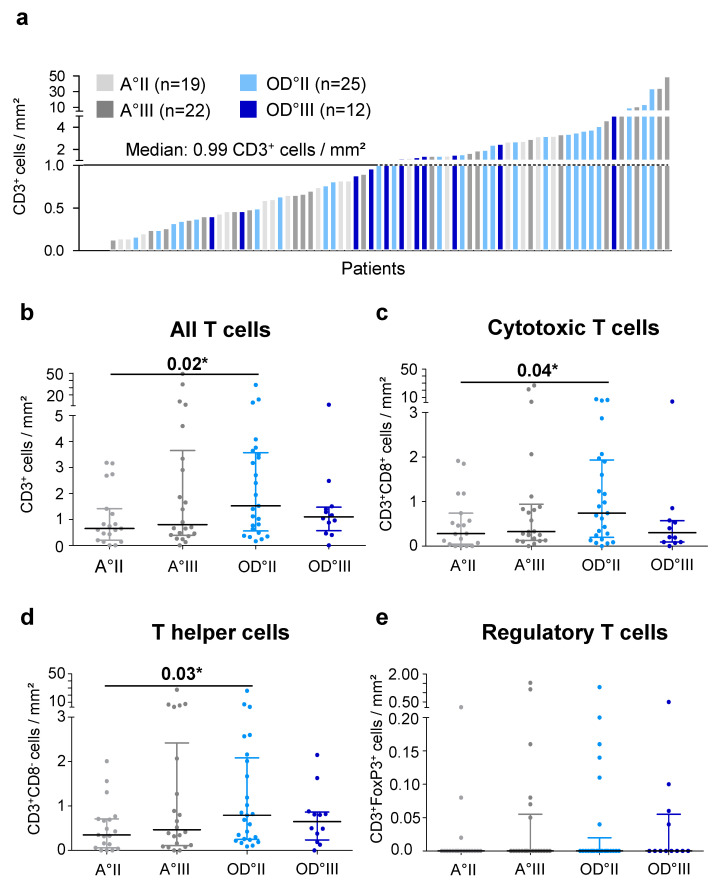
T cell infiltration in primary IDH^mut^ glioma: (**a**) distribution of T cell infiltration normalized to the area analyzed and color-coded for glioma subtype and WHO grade and (**b**–**e**) analysis of T cell infiltration in astrocytoma and oligodendroglioma categorized by their WHO grade for (**b**) all T cells, (**c**) cytotoxic T cells, (**d**) T helper cells, and (**e**) regulatory T cells. A, astrocytoma; OD, oligodendroglioma; mm^2^, square millimeter; II, WHO grade II; III, WHO grade III; * *p* < 0.05.

Detailed analysis of primary IDH^mut^ gliomas revealed a broad range of 0–48.97 CD3^+^ T cells/mm^2^ (median 0.99 T cells/mm^2^) (Figure 2a). Interestingly, when further stratifying our analysis for histological subtypes and WHO grades, we found a significantly higher T-cell infiltration (CD3^+^) in oligodendroglioma WHO grade II compared to astrocytoma WHO grade II (Figure 2b). This effect was also observed for cytotoxic and T helper cells but not for regulatory T cells (Figure 2c–e). However, a WHO grade-dependent change of T-cell infiltration could not be observed.

### 2.5. T-Cell Infiltration Increases in IDH^mut^ Recurrent Gliomas

**Figure 3:**
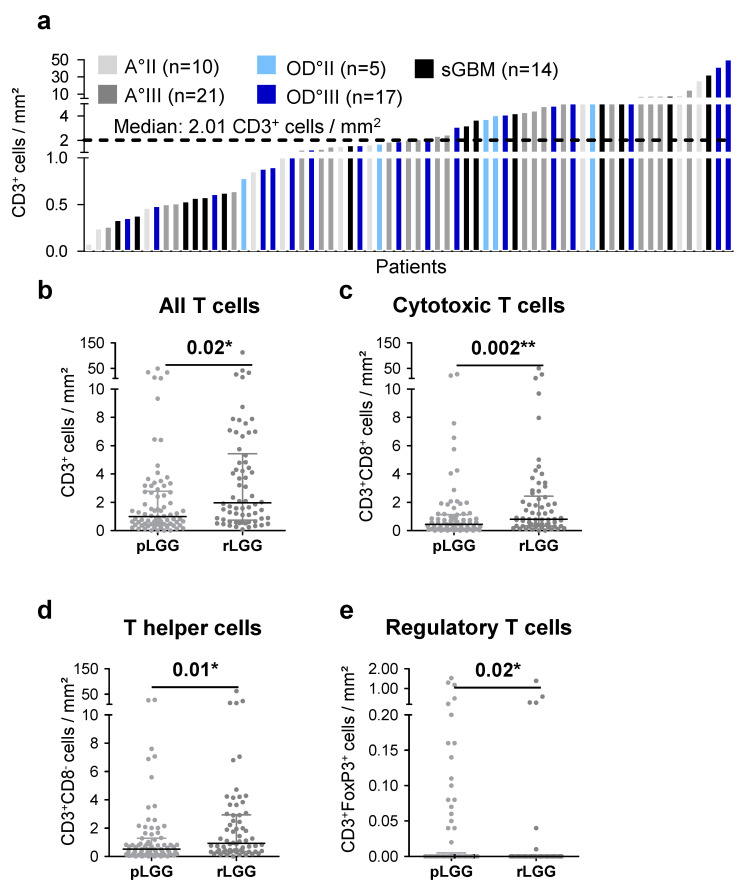
Changes in T-cell infiltration between primary and recurrent IDH^mut^ gliomas: (**a**) Distribution of T-cell infiltration in recurrent IDH^mut^ gliomas normalized to the area analyzed and color-coded for glioma subtype and WHO grade and (**b**–**e**) analysis of T-cell infiltration in primary and recurrent IDH^mut^ gliomas for (**b**) all T cells, (**c**) cytotoxic T cells, (**d**) T helper cells, and (**e**) regulatory T cells. A, astrocytoma; OD, oligodendroglioma, sGBM, secondary glioblastoma; mm^2^, square millimeter; II, WHO grade II; III, WHO grade III; pLGG, primary LGG; rLGG, recurrent LGG; * *p* < 0.05; and ** *p* < 0.01.

Because tumor evolution and treatment of the primary tumor may alter the type and rate of immune cell infiltration in recurrent tumors, we analyzed T-cell infiltration in an additional study sample of 66 recurrent IDH^mut^ gliomas (Table 2). Similar to primary IDH^mut^ gliomas, there was a highly variable infiltration rate. In addition, there were no differences associated with the histological subtype or WHO grade within recurrent tumors. However, a significantly increased infiltration of T cells (CD3^+^) could be observed in recurrent IDH^mut^ gliomas in comparison to primary IDH^mut^ tumors, with a median of 2.01 CD3^+^ cells/mm^2^ compared to 0.99 CD3^+^ cells/mm^2^ (Figure 2a and Figure 3a), respectively. This was also the case for cytotoxic and T helper cells (Figure 3c–d), while for regulatory T cells, the opposite effect was observed, resulting in a significantly reduced infiltration of regulatory T cells in recurrent glioma (mean 0.08 vs. 0.04, Figure 3e).

### 2.6. Pretreatment of Recurrent IDH^mut^ Glioma Impacts T-Cell Infiltration

**Figure 4:**
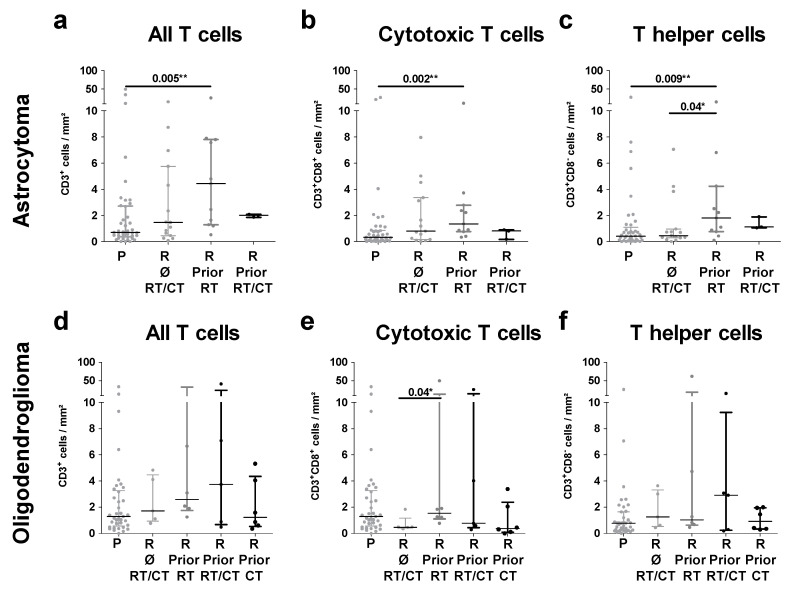
T-cell infiltration in primary and recurrent IDH^mut^ gliomas according to pretreatment: primary and recurrent (**a**–**c**) astrocytoma and (**d**–**f**) oligodendroglioma were classified according to the previous treatment of their predecessor tumor. Infiltration rates of (**a**,**d**) all T cells, (**b**,**e**) cytotoxic T cells, and (**c**,**f**) T helper cells were analyzed. P, primary; R, recurrent; Ø, no prior; RT, radiotherapy; CT, chemotherapy; RT/CT, radiochemotherapy; and mm^2^, square millimeter; * *p* < 0.05; and ** *p* < 0.01.

As tumor treatment is known to potentially impact the tumor microenvironment, we sought to investigate this hypothesis in our patient collective. To mirror the diverse therapies applied before the reoccurrence of an IDH^mut^ glioma, we divided our patient cohort into four groups: recurrent IDH^mut^ glioma without prior radio- or chemotherapy, recurrent IDH^mut^ glioma with prior radiotherapy, recurrent IDH^mut^ glioma with prior chemotherapy, and recurrent IDH^mut^ glioma with prior radio- and chemotherapy (Figure 4). The most pronounced effect was observed in patients with an astrocytoma that had received prior radiotherapy (Figure 4a–c). In recurrent astrocytoma, prior radiotherapy was associated with a significantly increased number of T cells in general (CD3^+^) as well as T helper and cytotoxic T cells in particular. However, in recurrent oligodendroglioma, this was only related to significantly higher numbers of cytotoxic T cells (Figure 4e). In contrast, prior treatment of recurrent tumors appeared to have no impact on the numbers of regulatory T cells (Figure S3).

### 2.7. Matched Pair Analysis Corroborates Beneficial Effects of Radiotherapy on T-Cell Infiltration

**Figure 5:**
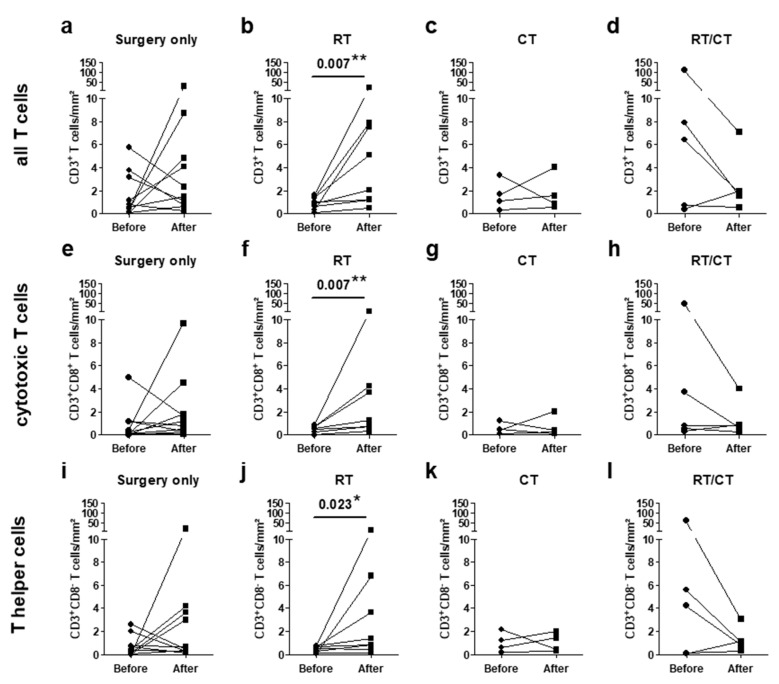
T-cell infiltration in pairs of IDH^mut^ glioma before and after adjuvant treatment: infiltration of (**a**–**d**) all T cells, (**e**–**h**) cytotoxic T cells, and (**i–l**) T helper cells were analyzed in pairs of glioma before and after the adjuvant treatment of the first tumor. Pairs were classified according to the pretreatment of the first tumor into (**a**,**e**,**i**) no adjuvant treatment (*n* = 10), (**b**,**f**,**j**) radiotherapy (*n* = 8), (**c**,**g**,**k**) chemotherapy (*n* = 4), and (**d**,**h**,**l**) radiochemotherapy (*n* = 6). RT, radiotherapy; CT, chemotherapy; RT/CT, radiochemotherapy; mm^2^, square millimeter; * *p* < 0.05; and ** *p* < 0.01.

To strengthen our hypothesis that previous radiotherapy might enhance T-cell infiltration, we reanalyzed cases of our study sample where either primary and recurrent IDH^mut^ gliomas or tumor tissues from two consecutive surgeries of the same patients were available. Accordingly, we identified 27 matched pairs. Of these, 19 pairs represented a combination of the primary tumor and its first recurrence, whereas the remaining eight matched pairs were obtained from the first and second recurrences. Again, the most impressive effect was observed in patients receiving prior radiotherapy only, with an increase of T cells as well as of helper and cytotoxic T cells (Figure 5). In contrast, T-cell infiltration in patients who did not receive radio- or chemotherapy or who received combined previous radiochemotherapy led to inconsistent results. In this case, we frequently observed a decrease in tumor-infiltrating T cells. Changes in T-cell infiltration of individual cases are illustrated in Figures S4–S6. Collectively, matched pair analysis strongly supports the assumption of a beneficial impact of prior radiotherapy on T-cell infiltration.

## 3. Discussion

In the present study, we analyzed T-cell infiltration in a substantial number of primary and recurrent IDH^mut^ gliomas by immunofluorescence staining followed by an objective counting of infiltrates in whole tissue slides and a software-based gating strategy. Through this reliable and robust measurement, we found a significantly higher T-cell infiltration in recurrent gliomas for T cells as well as for helper and cytotoxic T cells. Interestingly, this increased infiltration appears to be associated with the type of treatment of the primary tumor. Analysis of the entire study sample set as well as the subset of matched pairs displayed a consistent higher T-cell infiltration in recurrences when the preceding tumor was solely treated by radiotherapy. Altogether, this is the first study in IDH^mut^ gliomas focusing on immunological changes during tumor progression not only providing exact values of T-cell infiltration instead of semiquantitative scores but also supporting the idea of long-term changes in the microenvironment depending on the treatment of the primary tumor.

In line with previous findings, we further observed a lower T-cell infiltration in IDH^mut^ compared with IDH^wt^ astrocytoma. On a functional level, the latter has been attributed to production of the oncometabolite R-2-hydroxyglutarate by mutant IDH, which has been shown to impair T cell migration [6,16]. A further explanation might be the low mutational load of IDH^mut^ LGG and, thus, a low number of neoantigens capable of mounting T cell responses. Consequently, T cells are attracted less efficiently to the tumor site; hence, gliomas are regarded generally as immunologically cold tumors [19]. In addition, a study claims a more pronounced T_reg_ infiltration in astrocytoma [20], which was not observed in our study. One potential explanation might be that this study did not account for the IDH mutation status, and an unintentional inclusion of IDH^wt^ astrocytoma associated with a higher T-cell infiltration cannot be excluded. Thus, histology-associated differences of T-cell infiltration warrant further investigation of a considerable number of well-characterized cases.

One of our major findings consists of a substantial two-fold increase of T-cell infiltration in recurrent glioma, which specifically applied to cytotoxic and helper T cells. In a similar study comparing primary and recurrent gliomas, higher numbers of T cells appeared to be restricted to the perivascular niche of recurrent tumors [21]. However, information on the IDH status and an analysis of the impact of the adjuvant treatment was missing. Still, postsurgical treatment can be quite heterogeneous in gliomas, ranging from no adjuvant treatment to radio- and/or chemotherapy [7]. Therefore, we questioned whether a specific treatment history induced changes in the immune microenvironment, which are still present at the time of the recurrent tumor. This stratification revealed that only prior radiotherapy was associated with a consistently increased T-cell infiltration, while there were mixed results for all other treatment conditions. As local radiotherapy is generally regarded as activating the immune response by multifaceted effects, such as induction of immunogenic cell death, the release of tumor-associated antigens (TAAs) and danger-associated molecular patterns (DAMPs), promotion of dendritic cell activation, and cross-priming of naïve T cells, these functional consequences might be the underlying driving force for the observed radiation-associated increased T-cell infiltration [22,23]. However, so far, all these immunological changes in response to radiotherapy have been described as immediate effects while our analysis suggests the occurrence of radiation-induced long-term effects. Therefore, future studies should determine which of the known immediate immunological alterations are also responsible for the long-lasting changes of T-cell infiltration in the tumor microenvironment of recurrent glioma upon prior irradiation. However, why a combination of radio- and chemotherapy does not result in similar uniform T-cell infiltration changes in recurrent tumors remains another open question that should be explored in larger study samples using comprehensive molecular analysis. To date, there is considerable clinical data that combined radiochemotherapy causes lymphopenia, which, in turn, may lead to a lower T-cell infiltration from the periphery [24]. In contrast, alkylating chemotherapy has been shown to cause a hypermutator phenotype in recurrent tumors in a small subgroup of mismatch repair-deficient gliomas, resulting in increased T-cell infiltration [25,26]. Higher mutational loads are associated with increased numbers of intratumoral T cells, which might explain the opposing results we observed upon prior administration of chemotherapy [15,19]. Since this could have a profound impact on the efficacy of immunotherapy trials, a contribution of cytotoxic and cytostatic drugs to the long-lasting changes in T-cell infiltration should be analyzed in further detail in future studies.

In the current analysis, we used multicolor immunofluorescence staining in combination with a software-based cell counting on whole tissue slides to analyze a considerable number of primary and recurrent gliomas. Previous publications have primarily used immunohistochemistry of a single marker and manual cell counting in high-power fields (Table S1) [20,21]. The latter approach provides only limited and heavily biased information on T-cell infiltration and is even more critical when quantifying a low-abundant cell population such as T cells in glioma. In contrast, multicolor immunofluorescence staining enables multiparametric readouts from a single tissue section and, due to the utilized gating strategy, simultaneous analysis of different marker combinations. Accordingly, our setup appears to have some important advantages for the quantification of T-cell infiltration in IDH^mut^ glioma.

In summary, we report long-lasting changes in the tumor environment of IDH^mut^ gliomas. A consistent increase of helper and cytotoxic T cells in recurrent glioma in association with prior radiotherapy may have profound implications for future therapy decisions, therefore warranting further investigations to explore the underlying mechanisms.

## 4. Materials and Methods

### 4.1. Patients and Specimens

Primary and recurrent glioma specimens (*n* = 156) were obtained from patients who underwent surgical resection at the Department of Neurosurgery, University Hospital of Heidelberg (Table 1 and Table 2 and Tables S2 and S3). A tumor cell content ≥60% and absence or presence of the IDH1(R132H) mutation was assessed for all samples by experienced neuropathologists (A.v.D. and D.R.). The use of patient material was approved by the Heidelberg Medical Faculty Review Board, in accordance with the Declaration of Helsinki. Written informed consent was obtained from all patients. Samples used for the preparation of cryosections and immunofluorescence staining were snap-frozen immediately after surgery and stored at −80 °C.

### 4.2. Preparation of Glioma Cryosections

Cryosections from tumor samples were prepared using the cryostat Leica CM3050. Tissue samples were embedded in the medium Tissue-Tek^®^ O.C.T.™ (Sakura, Alphen aan den Rijn, The Netherlands) at a cryostat chamber temperature of −25 °C, cut into 5- to 7-µm thick sections, and rapidly mounted at room temperature on adhesion microscope slides. To evaluate tissue preservation and orientation, methylene blue staining was performed. Additionally, slides were stained with hematoxylin and eosin (H&E) for further tumor quality evaluation. The prepared slides were dried overnight, acetone-fixed for 10 min at −20 °C, and stored at −80 °C until further use.

### 4.3. Multicolor Immunofluorescence Staining and Data Acquisition

Quantification of T cell subpopulations was conducted by a combination of the primary antibodies anti-CD3 (polyclonal, Dako, Santa Clara, California, United States), anti-CD8 (YTC182.2, Abcam, Cambridge, United Kingdom), and anti-FoxP3 (236A/E7, Abcam) (Table S4). Primary antibodies were diluted in DAKO Diluent, incubated for 60 min in a humidified chamber, and washed three times for 5 min in 1× PBS + 0.05 % Tween^®^ 20. Secondary antibodies (all from Life Technologies, Carlsbad, California, United States, Table S5) were diluted in 1× Gibco^®^ DPBS, incubated in a dark humidified chamber for 60 min, and washed three times for 5 min in PBS. To stain cell nuclei, DAPI (Life Technologies) was diluted in PBS and incubated with the secondary antibody solution. All multicolor immunofluorescence staining was performed on cryosections and complemented by isotype-matched (Table S6) and negative controls (without primary antibodies). Entire tissue sections were scanned on an Olympus IX51 microscope equipped with a XM10 camera (both Olympus, Hamburg, Germany) and analyzed by the TissueQuest Cell Analysis software package (version 4.0.1.0137, TissueGnostics GmbH, Vienna, Austria). For automated analysis with TissueQuest, DAPI staining was used as a master marker for cell identification based on nuclei detection. Regions of interest were manually defined to distinguish between tumor and adjacent infiltration zone based on H&E staining approved by board-certified neuropathologists (A.v.D. and D.R.). All tissues were analyzed with identical cell detection parameters based on nuclear size, cell compactness, and cell eccentricity. Appropriate events were then gated according to the mean CD3 fluorescence intensity. CD3^+^ events were further separated into CD8^+^ and CD8^−^ as well as into FoxP3^+^ and FoxP3^−^ cells based on the mean staining intensity of those markers. Finally, all cells detected were validated manually by backward gating on the original image and visualized as scattergrams.

### 4.4. Statistical Analysis

Data were analyzed using the software GraphPad Prism 5.03 (GraphPad Software Inc., San Diego, California, United States). Since T-cell counting data are often not normally distributed, statistical significance was determined using the unpaired Mann–Whitney U test. Results with a *p* value < 0.05 were defined as statistically significant (* *p* < 0.05, ** *p* < 0.01, and *** *p* < 0.001). Whiskers depict the median ± interquartile range.

## Figures and Tables

**Table 1:** Clinical data of patients with IDH-mutant (IDH^mut^) primary lower-grade glioma.

Variable	*n* = 78	Patients (%)	Mean (Range)
Sex			
Male	45	57.69	
Female	33	42.31	
Age ^1^			43.24 (20.77–80.06)
WHO grade			
WHO grade II	44	56.41	
Astrocytoma	19	43.18	
Oligodendroglioma	25	56.82	
WHO grade III	34	43.59	
Astrocytoma	22	64.71	
Oligodendroglioma	12	35.29	

^1^ At initial diagnosis (years).

**Table 2:** Clinical overview of patients with IDH^mut^ recurrent glioma.

Variable	*n* = 66	Patients (%)	Mean (Range)
Sex			
Male	42	63.63	
Female	24	36.36	
Age ^1^			42.24 (20.76–71.45)
WHO grade			
WHO grade II	15	22.72	
Astrocytoma	10	66.67	
Oligodendroglioma	5	33.33	
WHO grade III	37	56.06	
Astrocytoma	20	54.05	
Oligodendroglioma	17	45.95	
WHO grade IV			
Glioblastoma	14	21.21	
Treatment of previous tumor			
Surgery only	23	34.85	
Astrocytoma	15	65.22	
Oligodendroglioma	5	21.74	
Glioblastoma	3	13.04	
Radiotherapy	22	31.82	
Astrocytoma	12	52.38	
Oligodendroglioma	6	28.57	
Glioblastoma	4	19.05	
Chemotherapy	8	12.12	
Astrocytoma	1	12.50	
Oligodendroglioma	6	75.00	
Glioblastoma	1	12.50	
Radiochemotherapy	14	21.21	
Astrocytoma	3	21.43	
Oligodendroglioma	5	35.71	
Glioblastoma	6	42.86	

^1^ At initial diagnosis (years).

## Supplementary Materials

Supplementary materials can be found at https://www.mdpi.com/1422-0067/21/20/7801/s1. Figure S1: Gating strategy identifying tumor infiltrating lymphocytes in whole tissue sections using the software TissueQuest; Figure S2: TIL infiltration rates in IDHwt and IDHmut LGG; Figure S3: Infiltration rate of regulatory T cells in primary and recurrent IDHmut LGG according to the pretreatment; Figure S4: Overall T-cell infiltration rate in paired IDH^mut^ LGG categorized according to the treatment of first tumor; Figure S5: Cytotoxic T-cell infiltration rate in paired IDH^mut^ LGG categorized according to the treatment of first tumor; Figure S6: T helper cell infiltration rate in paired IDH^mut^ LGG categorized according to the treatment of first tumor; Table S1: Literature overview; Table S2. Progression free survival of patients with IDH^mut^ primary lower-grade glioma; Table S3: Clinical data of patients with IDH1^wt^ primary glioma; Table S4: Primary anti-human antibodies; Table S5: Secondary antibodies; Table S6: Isotype control antibodies.

## Abbreviations

A	astrocytoma
CT	chemotherapy
GBM	Glioblastoma
IDH	isocitrate dehydrogenase
IDH^mut^	IDH-mutant
IDH^wt^	IDH-wildtype
LGG	lower-grade glioma
OD	oligodendroglioma
pLGG	primary LGG
rLGG	recurrent LGG
RT	radiotherapy
RT/CT	radiochemotherapy
sGBM	secondary glioblastoma
