# Supplementary material for: Increased Radiation-Associated T-Cell Infiltration in Recurrent IDH-Mutant Glioma
Source: Int J Mol Sci. 2020 Oct 21;21(20):7801. doi: 10.3390/ijms21207801 (PMC7590222; doi:10.3390/ijms21207801)
Supplement: Supplementary file 1 [file ijms-21-07801-s001.pdf]

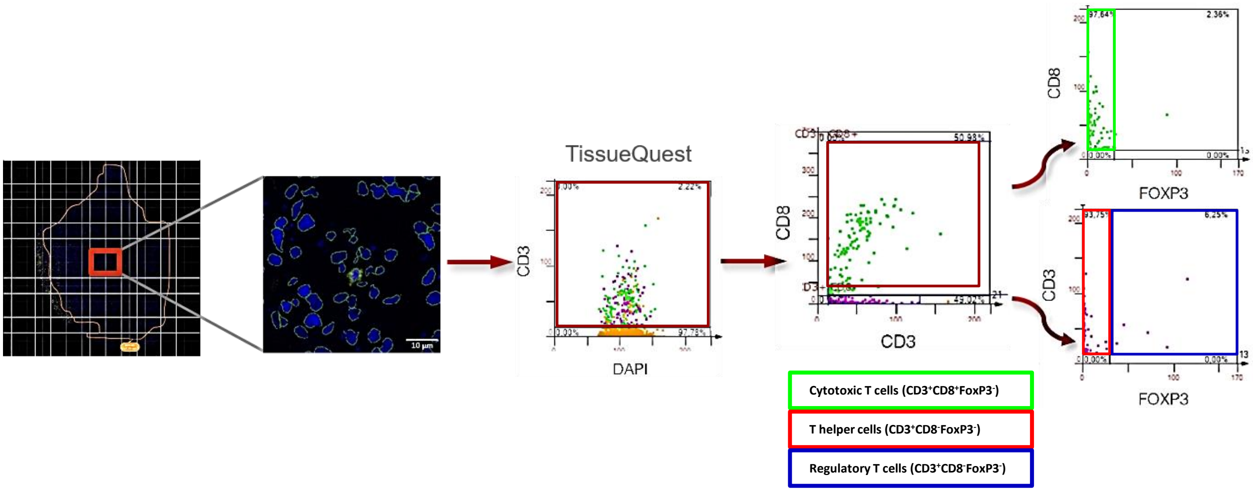

Figure S1: Gating strategy identifying tumor infiltrating lymphocytes in whole tissue sections using the software TissueQuest. Exemplary representation of flow cytometry-like scatter diagrams quantifying cytotoxic T cells (CD3+CD8+FOXP3-), T helper cells (CD3+CD8-FOXP3-), and regulatory T cells (CD3+CD8-FOXP3+).

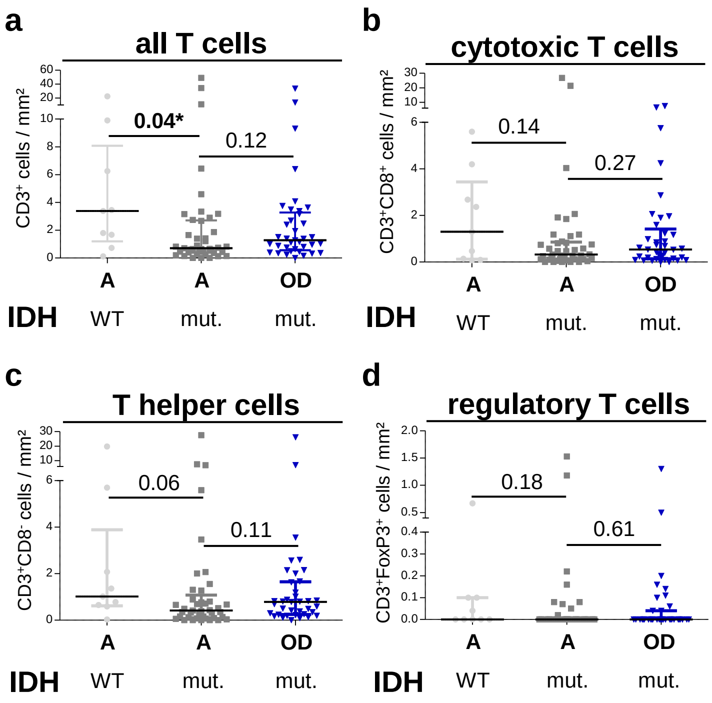

Figure S2: TIL infiltration rates in IDH^{wt} and IDH^{mut} LGG. Comparison of **(a)** all T cells, **(b)** cytotoxic T cells, **(c)** T helper cells and **(d)** regulatory T cells between IDH wild-type astrocytoma and IDH mutated astrocytoma/oligodendroglioma normalized to square millimeter. A, astrocytoma; OD, oligodendroglioma; WT, IDH-wild-type; mut., IDH mutated.

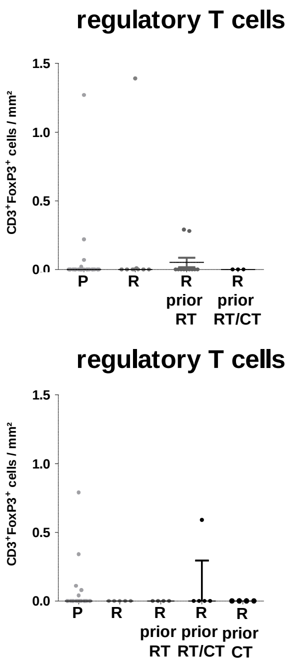

Figure S3: Infiltration rate of regulatory T cells in primary and recurrent IDHmut LGG according to the pretreatment. p, primary; r, recurrent; RT, radiotherapy; CT, chemotherapy; RT/CT, radiochemotherapy; mm^2, square millimeter.

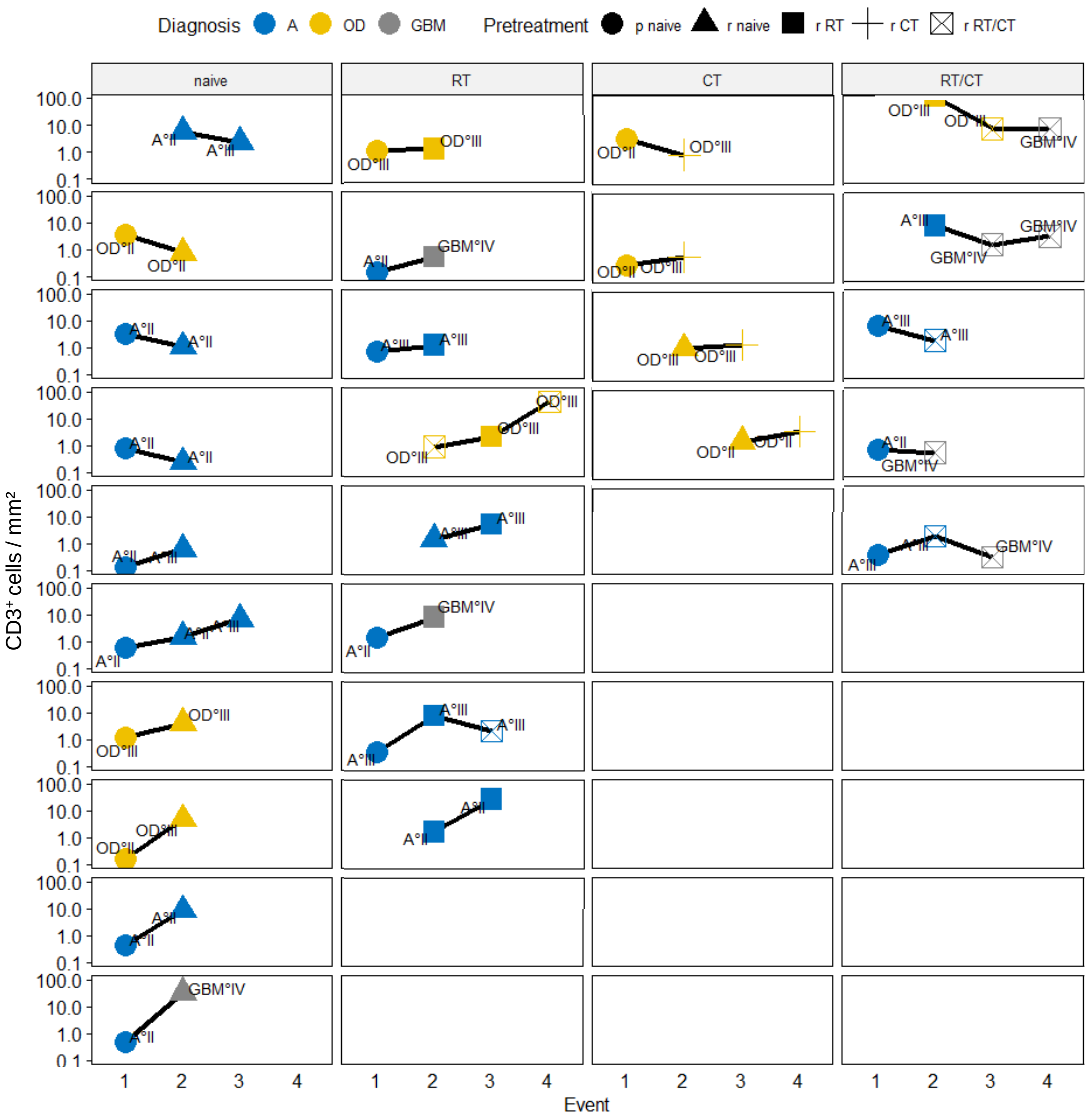

Figure S4: Overall T cell infiltration rate in paired IDH^{mut} LGG categorized according to the treatment of first tumor. Each box shows an individual patient and the numbers represent the tumor events starting from with 1 as the initial glioma. Naïve, surgery only; RT, radiotherapy; CT, chemotherapy; RT/CT, radiochemotherapy; mm^2, square millimeter; A, astrocytomas; OD, oligodendrogliomas, GBM, glioblastoma.

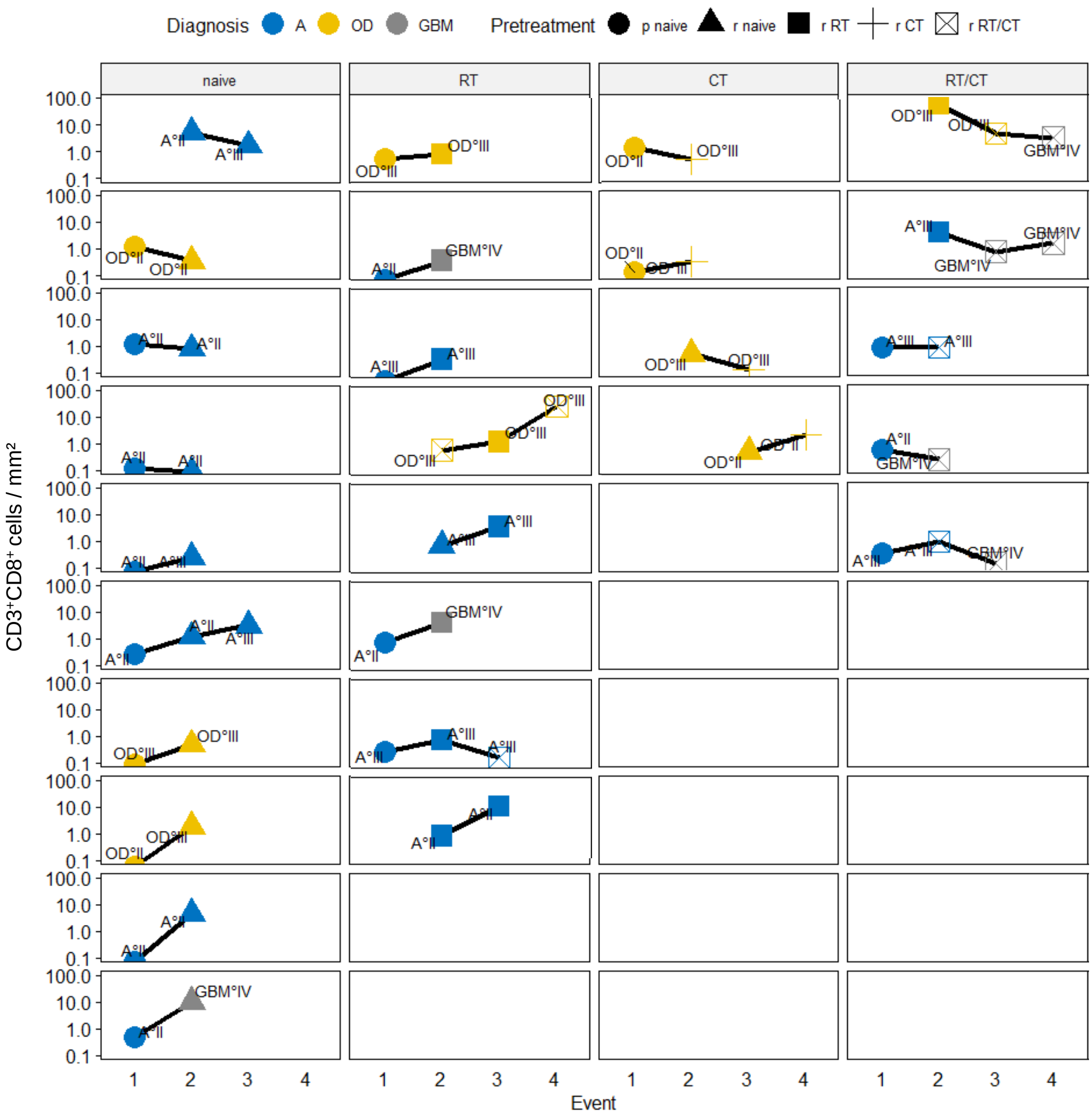

Figure S5: Cytotoxic T cell infiltration rate in paired IDH^{mut} LGG categorized according to the treatment of first tumor. Each box shows an individual patient and the numbers represent the tumor events starting from with 1 as the initial glioma. Naïve, surgery only; RT, radiotherapy; CT, chemotherapy; RT/CT, radiochemotherapy; mm^2, square millimeter A, astrocytomas; OD, oligodendrogliomas, GBM, glioblastoma.

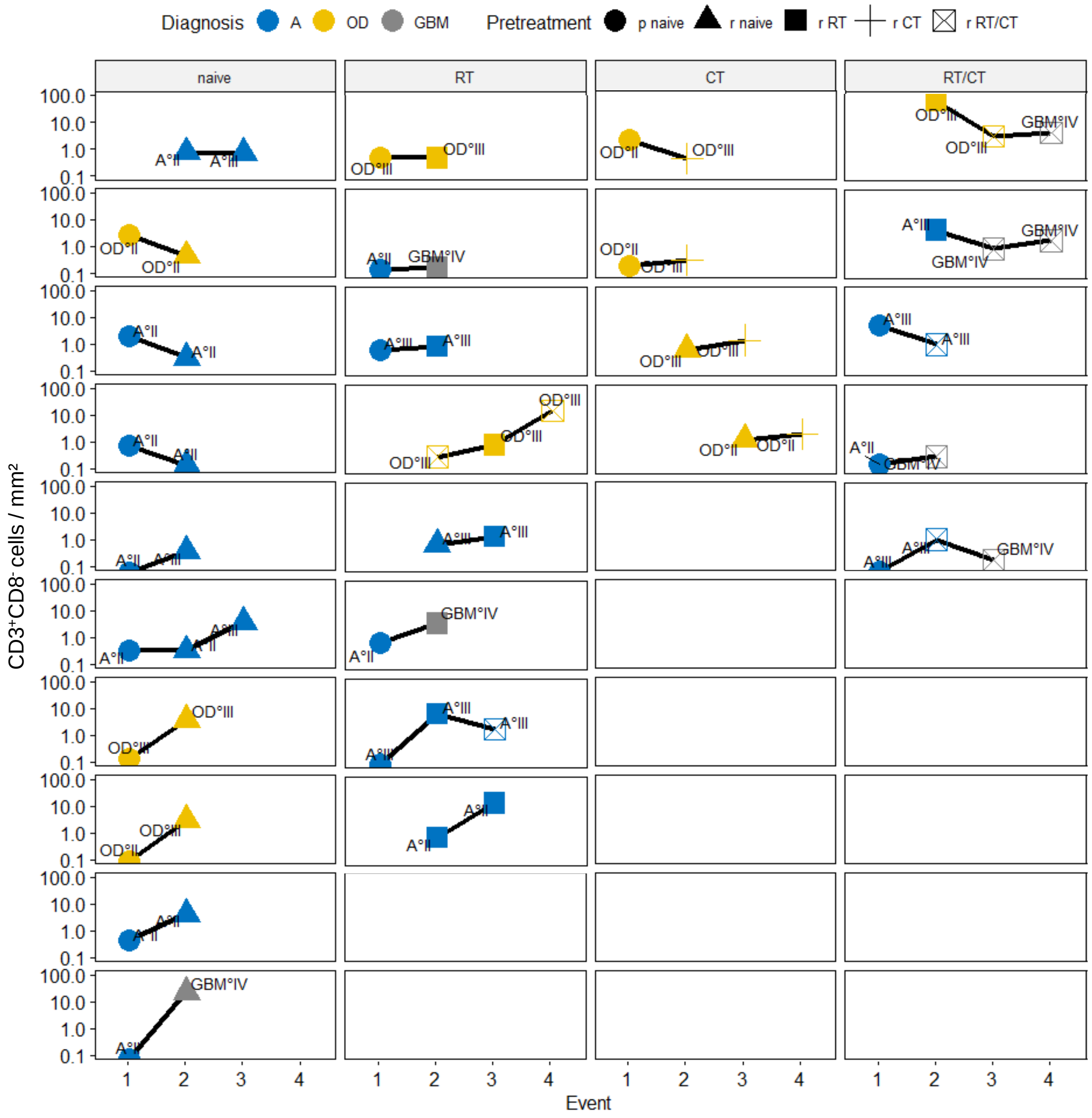

Figure S6: T helper cell infiltration rate in paired IDH^{mut} LGG categorized according to the treatment of first tumor. Each box shows an individual patient and the numbers represent the tumor events starting from with 1 as the initial glioma. Naïve, surgery only; RT, radiotherapy; CT, chemotherapy; RT/CT, radiochemotherapy; mm^2, square millimeter A, astrocytomas; OD, oligodendrogliomas, GBM, glioblastoma.

Table S1: Literature overview

**Author, publication year**	**Journal**	**Main topic**	**Technique**	**Study sample**	**Study sample IDH^{mut} tumors**	**Discrimination PT vs. RT**	**Treatment analysis**
Berghoff et al., 2017	Neuro-Oncology	Differences in TIL infiltration and PD-L1 expression in IDH^{mut} and IDH^{wt} glioma	Immunohistochemistry for CD3 expression; data presentation as presence or absence of CD3 positive cells estimated in 100x magnification	**IDH^{mut}**: 22x A, 17x O; 14x GBM<br>**IDH^{wt}:** 4x A,	**N= 57**	**no**	**no**
Bunse et al., 2018	Nature medicine	Functional impact of 2-HG on T cells in IDH^{mut} glioma	Immunohistochemistry for CD4 or CD8 protein expression; analysis for presence or absence of CD4 or CD8 positive cells in 100x magnification	**IDH^{mut}**<br>-CD4: 22x A, 12x O, 24x OA<br>-CD8: 36x A, 22x O, 31x OA<br>**IDH^{wt}**<br>-CD4: 10x A, 1x O, 5x OA<br>-CD8: 14x A, 2x O, 7x OA	**CD4<br>N=58<br><br>CD8<br>N=89**	**60% PT, rest undefined**	**no**
Kohanbash et al., 2017	JCI	Functional impact of 2-HG on T cells in IDH^{mut} glioma	3 color immune fluorescence staining, software based quantitative assessment of entire tissue slices (200x magnification)	**IDH^{mut}:** 11x A,<br>**IDH^{wt}:** 9x A	**N=11**	**no**	**no**
Zhang et al., 2018	CCR	2-HG in IDH^{mut} gliomas inhibits complement and T cells	Immunohistochemistry for CD4, CD8 or FOXP3; cell counting in about 10 high power fields at 400x magnification	**IDH^{mut}:** 23x A, 14x GBM<br>**IDH^{wt}:** 16x A, 19x GBM	**N=37**	**PT**	**no**
Makarevic et al. 2020	Current study (IJMS)	Therapy-associated T cell infiltration in primary and recurrent IDH^{mut} glioma	4 color immune fluorescence staining, software based quantitative assessment of entire tissue slices (200x magnification)	**IDH^{mut}**: 71x A, 59x O; 14x sGBM<br><br>**IDH^{wt}:** 9x A, 4x GBM	**Primary<br>N=78<br><br>Recurrent<br>N=66**	**yes**	**yes**

A = astrocytoma; O = oligodendroglioma; OA = oligoastrocytoma; GBM = glioblastoma; PT = primary tumor; RT = recurrent tumor

Table S2. Progression free survival of patients with IDH^{mut} primary lower-grade glioma.

Variable	n = 78	Median progression free survival [months]	range
**WHO grade II**			
Astrocytoma	19	44.0	6.0 – 79.5
Oligodendroglioma	25	108.0	2.0 – 219.6
**WHO grade III**			
Astrocytoma	22	26.6	5.5 – 208.0
Oligodendroglioma	12	77.0	14.8 – 236.0

Table S3: Clinical data of patients with IDH1^{wt} primary glioma

Variable			n = 12	Patients [%]	Mean [range]
**Sex**					
		Male	**8**	57.14	
		Female	**5**	38.46	
**Age**[1]					50.63 [15.92-75.68]
**WHO grade**					
	**WHO grade II**	Astrocytoma	**2**	15.38	
	**WHO grade III**	Astrocytoma	**7**	53.85	
	**WHO grade IV**	Glioblastoma	4	30.77	

[1] At initial diagnosis [years]

Table S4: Primary anti-human antibodies

Antigen	Clone	Host spezies	IgG-Subtype	Source
**CD3**	polyclonal	rabbit	IgG	DAKO
**CD8**	YTC182.2	rat	IgG2b	Abcam
**FoxP3**	236A/E7	mouse	IgG1	Abcam

Table S5: Secondary antibodies

Antibody	Specificity	Host spezies	Source
**Alexa Fluor® 647**	anti-rabbit	goat	Invitrogen
**Alexa Fluor® 488**	anti-rat	donkey	Invitrogen
**Alexa Fluor® 555**	anti-mouse	goat	Invitrogen

Table S6: Isotype control antibodies

Antigen	Host spezies	Source
**Isotype IgG**	rabbit	OriGene Europe
**Isotype IgG1**	mouse	OriGene Europe
**Isotype IgG2b**	rat	OriGene Europe
